# The Use of Risperidone in Behavioral and Psychological Symptoms of Dementia: A Review of Pharmacology, Clinical Evidence, Regulatory Approvals, and Off-Label Use

**DOI:** 10.3389/fphar.2020.00596

**Published:** 2020-05-20

**Authors:** Ismaeel Yunusa, Marie Line El Helou

**Affiliations:** ^1^School of Pharmacy, Massachusetts College of Pharmacy and Health Sciences, Boston, MA, United States; ^2^School of Pharmacy, Lebanese American University, Byblos, Lebanon

**Keywords:** behavioral and psychological symptoms of dementia, risperidone, Alzheimer’s disease, review (article), dementia

## Abstract

Dementia represents a global health challenge due to the increase in elderly population worldwide. In addition to memory loss, dementia often results in severe behavioral and psychological changes where pharmacological treatments might be considered in addition to nonpharmacological strategies for optimal symptomatic control. Risperidone, the second oldest atypical antipsychotic, has been widely used off-label to treat behavioral and psychological symptoms of dementia (BPSD), including agitation, aggression, and psychosis. Several studies have indicated that risperidone offers a modest and statistically significant effectiveness in the clinical setting. However, in the past decade, safety concerns emerged due to increased risk for cerebrovascular adverse events and death following the use of risperidone in the elderly population. Clinical guidelines suggest that, in severe dementia where an older adult is threatening to harm himself or others, pharmacological treatments might be considered when nonpharmacological treatments fail. Risperidone was approved for BPSD in some countries (Australia, Canada, United Kingdom and New Zealand) but not in the United States. This article reviews risperidone’s pharmacological activity, clinical effectiveness and safety, marketing approval, and off-label use in BPSD.

## Introduction

More than 40 million people live with dementia worldwide, and this number is projected to triple by 2050 (Prince et al., 2015). It occurs more commonly in persons 65 or older, and with the growing elderly population in developed countries, dementia represents a global health challenge (Plassman et al., 2007; Livingston et al., 2017). Dementia is characterized by progressive memory decline with different underlying causes, including Alzheimer’s disease, vascular dementia, and Lewy bodies, among others. In addition to memory loss, behavioral symptoms common to dementia which are thought to be targets for antipsychotic drugs include: declined ability to solve problems, difficulty in maintaining emotional control, agitation, aggression, delusion, apathy, impulsivity, depression, and hallucinations (Dementia Society of America, 2019). Behavioral and psychological symptoms of dementia (BPSD) add to the burden of the disease and further reduce the quality of life of the patients and their caregivers (Anderson et al., 2019).

Acetylcholinesterase inhibitors (galantamine, donepezil, rivastigmine) and memantine are the mainstay treatment for dementia-related cognitive symptoms. The first-line treatment of BPSD are nonpharmacological treatments including environmental (e.g., use of familiar objects) and social techniques (redirection and frequent re-orientation). However, medications like antidepressant and antipsychotics are sometimes considered as treatment options at later stages for optimal management of severe BPSD (Motsinger et al., 2003; Bessey and Walaszek, 2019; Ringman and Schneider, 2019). Among antipsychotics, atypical drugs (including clozapine, risperidone, olanzapine, aripiprazole, quetiapine, etc.) are favored over typical due to less extrapyramidal side effects (EPS). Although, clozapine is the first atypical antipsychotic to be approved for other indications; it did not gain widespread use over risperidone because of its increased risk of agranulocytosis (Liperoti et al., 2008). Moreover, among all the atypical antipsychotics, risperidone has the most clinical trial-related evidence to support its use in BPSD (Lee et al., 2006).

In this review, we will focus on the role of risperidone, one of the oldest and most widely used atypical antipsychotics in the management of BPSD. We will first review risperidone’s pharmacological activity, secondly, we will explore the clinical evidence behind its use in BPSD and finally, we will examine its worldwide regulatory approval and off-label use in BPSD.

## Pharmacology of Risperidone

Risperidone is a benzisoxazole derivative. It was the second atypical antipsychotic developed following clozapine. It quickly became a first-line treatment for schizophrenia because of its favorable side effect profile compared to clozapine (Chopko and Lindsley, 2018). Like other atypical agents, its antipsychotic activity is believed to be linked primarily to central antagonism of dopamine-2 (D2) and serotonin-2C (5HT2C) receptors (Love and Nelson, 2000). Compared to conventional antipsychotics like haloperidol, risperidone bocks 5-HT2 receptors with a higher affinity than D2 receptors. The blockade of 5-HT2A receptors is thought to confer increased dopaminergic transmission in the nigrostriatal pathway and hence results in reduced risk of EPS compared to conventional antipsychotics (Álamo and López-Muñoz, 2013). However, in clinical practice, it should be noted that risperidone can cause EPS in a dose-dependent manner, especially with doses above 6 mg/d (Motsinger et al., 2003; Calsolaro et al., 2019). While EPS is a grossly overused and poorly understood term, risperidone-related EPS such as akathisia and parkinsonism are extremely under-recognized in elderly patients with dementia (Thomson et al., 2017; Duma and Fung, 2019).

Moreover, risperidone blocks α1 and α2 adrenergic receptors as well as H1 histaminergic receptors, which contributes to other pharmacological properties (FDA, 2009). Antagonism of α2 adrenergic receptors is believed to contribute to antidepressant activity and blockade of H1 receptors leads to sedation. Antagonism of α1 adrenergic receptors accounts for orthostatic hypotension. Also, several lines of evidence suggest that antagonizing 5-HT2C receptors results in antidepressant and anxiolytic properties. Risperidone has little or no in vitro affinity on cholinergic muscarinic receptors, which is translated in clinical practice as minimal anticholinergic side effects (Jensen et al., 2010). This is of particular interest in the elderly population where anticholinergic burden (risk of fall, delirium, and confusion) is an important safety concern.

Risperidone has a fast onset of action due to its rapid gastrointestinal absorption and quickly reaches a steady-state plasma concentration due to its short half-life (2.8 h). This property favors its use for the management of severe acute psychosis (Claus et al., 2006; Álamo and López-Muñoz, 2013), with similar pharmacological properties and a significantly longer long half-life (24.8 h) is responsible for the extended duration of action, which allows for a once-daily administration (Mauri et al., 2014).

## Clinical Use of Risperidone in Dementia-Related Psychosis, Aggression, and Agitation

**Table 1** presents a summary of findings from notable systematic reviews and meta-analyses of clinical trials that compared risperidone with other atypical antipsychotics or placebo. Clinical evidence from systematic literature reviews and meta-analyses suggests that risperidone as other atypical antipsychotics, provides a small but statistically significant benefit compared to placebo in treating psychosis, aggression, and agitation in individuals with dementia. However, like other antipsychotics, risperidone is accompanied by potentially serious adverse effects. These include, parkinsonism, falls, an increased risk of death, and cerebrovascular adverse events (CVAE [including stroke and transient ischemic attack]) (Maher et al., 2011; Tampi et al., 2016). An increase in the risk of CVAE and deaths with antipsychotics in the elderly population has been widely reported in the literature and resulted in a boxed warning by the US Food and Drug Administration (FDA) placed on risperidone’s package as well as all other antipsychotics’ package (Chatterjee et al., 2012). Some studies have argued that the risk of CVAE in patients with BPSD is overestimated and evidence came from patients with predisposing risks such as underlying vascular type of dementia, previous stroke, and insufficient management of hypertension (Herrmann and Lanctôt, 2005; Shin et al., 2013). However, according to a network meta-analysis (NMA) comparing different atypical antipsychotics (risperidone, olanzapine, aripiprazole, and quetiapine) for the treatment of BPSD, no statistically significant difference was seen between antipsychotics in terms of effectiveness, death, or CVAE (Yunusa et al., 2019). This NMA found that risperidone only provided a superior improvement over placebo on Cohen-Mansfield Agitation Inventory (CMAI) scale while aripiprazole improved Neuropsychiatric Inventory and Brief Psychiatric Rating Scale in addition to CMAI (Overall and Gorham, 1962; Cummings et al., 1994; Cohen-Mansfield, 1996; Wood et al., 2000). In this NMA, simultaneous ranking of effectiveness and safety suggests that aripiprazole could be a safer treatment option in patients with a history of stroke or other risk factors of CVAE.

**Table 1:** Findings from some notable systematic reviews and meta-analyses comparing risperidone with other atypical antipsychotics or placebo (Schneider et al., 2006; Schneider et al., 2005; Maher et al., 2011; Farlow and Shamliyan, 2017; Jin and Liu, 2019; Yunusa et al., 2019).

Study	Study Type	Study objective	Comparison^a^	Summary of Findings	
				Efficacy	Safety^b^
Schneider et al., 2006	SR/MA	Assess the evidence for efficacy and adverse events of atypical antipsychotics for people with dementia.	Risperidone vs Placebo	Behave-AD; risperidone significantly improved Behave-AD compared with placebo (WMD, −1.48; 95% CI, −2.35, −0.61; 4 studies). CMAI; risperidone significantly improved CMAI compared with placebo (WMD, −3.00; 95% CI, −4.22, −1.78; 3 studies) BPRS; risperidone did not significantly improve BPRS compared with placebo (WMD, 0.60; 95% CI, −1.82, 3.02; 1 study). NPI; risperidone did not significantly improve NPI compared with placebo (WMD, 2.60; 95% CI, −2.70, 7.90; 1 study). CGI-S; risperidone did not significantly improve CGI-S compared with placebo (WMD, −0.09; 95% CI, −0.21, 0.02; 3 studies).	CVAE; There was a significant increase in the risk of CVAE for risperidone compared with placebo (OR, 3.43; 95% CI, 1.60, 7.32; 4 studies). Death; There was no significant increase in the risk of death for risperidone compared with placebo (OR, NR; 95% CI, NR; 5 studies).
Schneider et al., 2005	SR/MA	Assess the evidence for increased mortality from atypical antipsychotics for people with dementia.	Risperidone vs Placebo	N/A	CVAE; N/A Death; There was no significant increase in the risk of death for risperidone compared with placebo (OR, 1.30; 95% CI, 0.76, 2.23; 5 studies).
Maher et al., 2011	SR/MA	Assess the efficacy and safety of atypical antipsychotic medications for use in conditions lacking approval for labeling and marketing by the US Food and Drug Administration.	Risperidone vs Placebo	Global score^c^; risperidone did not significantly improve global scores compared with placebo (SMD, 0.19; 95% CI, 0, 0.38; 6 studies).	CVAE; There was a significant increase in the risk of CVAE for risperidone compared with placebo (OR, 3.12; 95% CI, 1.32, 8.21; 4 studies). Death; NR
Farlow and Shamliyan, 2017	SR/MA	Assess the efficacy and safety of atypical antipsychotics for people with dementia.	Risperidone vs Placebo	Behave-AD; risperidone did not significantly improve Behave-AD compared with placebo (SMD, −0.20; 95% CI, −0.40, 0.00; 4 studies). BPRS; risperidone did not significantly improve BPRS compared with placebo (SMD, −0.10; 95% CI, −0.60, 0.30; 2 studies). NPI; risperidone did not significantly improve NPI compared with placebo (SMD, −0.10; 95% CI, −0.60, 0.40; 2 studies). CGI; risperidone significantly improved CGI-T compared with placebo (SMD, −0.40; 95% CI, −0.70, −0.10; 4 studies).	CVAE; There was a significant increase in the risk of CVAE for risperidone compared with placebo (OR, 4.53; 95% CI, 1.75, 11.72; 4 studies). Death; There was no significant increase in the risk of death for risperidone compared with placebo (RR, 3.70; 95% CI, 0.20, 88.5; 1 study).
Yunusa et al., 2019	SR/NMA	Assess the relative benefits and safety of atypical antipsychotics in the treatment of BPSD shown in randomized clinical trials using network meta-analysis.	Risperidone vs Placebo vs Aripiprazole vs Olanzapine vs Quetiapine	CMAI; There was no statistically significant difference between risperidone and other atypical antipsychotics based on CMAI. Risperidone significantly improved CMAI compared with placebo (SMD, −0.30; 95%CI, −0.55, −0.05) BPRS; There was no statistically significant difference between risperidone and other atypical antipsychotics based on BPRS. Risperidone did not significantly improved BPRS compared with placebo (SMD, −0.10; 95% CI, −0.29, 0.09). NPI; There was no statistically significant difference between risperidone and other atypical antipsychotics based on NPI. Risperidone did not significantly improved NPI compared with placebo (SMD, −0.01; 95% CI, −0.19, 0.18).	CVAE; There was no statistically significant difference between risperidone and other atypical antipsychotics based on CVAE.There was a significant increase in the risk of CVAE for risperidone compared with placebo (OR, 3.85; 95% CI, 1.55, 9.55). Death; There was no statistically significant difference between risperidone and other atypical antipsychotics based on mortality. No significant increase in the risk of death for risperidone compared with placebo (RR, 1.32; 95% CI, 0.77, 2.27).
Jin and Liu, 2019	SR/NMA	Assess the comparative efficacy and safety of pharmacological and nonpharmacological therapies for the BPSD.	Risperidone vs Placebo vs Aripiprazole vs Olanzapine vs Quetiapine vs Haloperidol vs Other antipsychotic agents	CMAI; There was no statistically significant difference between risperidone and other atypical antipsychotics based on CMAI. Risperidone significantly improved CMAI compared with placebo (MD, −2.58; 95% CrI, −5.20, −0.60) NPI; There was no statistically significant difference between risperidone and other atypical antipsychotics based on NPI. Risperidone did not significantly improved NPI compared with placebo (MD, −3.20; 95% CrI, −6.08, −0.31).	CVAE; There was no statistically significant difference between risperidone and other atypical antipsychotics based on CVAE. There was a significant increase in the risk of CVAE for risperidone compared with placebo (OR, 3.94; 95% CrI, 1.85, 10.73). Death; NR

BPDS, behavioral and psychological symptoms of dementia; BPRS, Brief Psychiatry Rating Scale; CGI, Clinical Global Impression (total score); CGI-S, Clinical Global Impression – Severity scale; CMAI, Cohen-Mansfield Agitation Inventory; CVAE, cerebrovascular adverse events; CI, confidence interval; CrI, credible interval; MD, mean difference; N/A, not applicable or not available; NMA, network meta-analysis; NR, not reported; NPI, neuropsychiatry inventory; OR, odds ratio; RR, relative risk; SR/MA, systematic review and meta-analysis (pairwise); SR/NMA, systematic review and network meta-analysis; SMD, standardized mean difference; WMD, weighted mean difference.

a Refers to only comparisons involving risperidone.

b Safety outcomes limited to death and cerebrovascular adverse events for which there are regulatory warnings across the world.

c Refers to a total global score that included cumulative psychiatric symptoms of delusions, hallucinations, suspiciousness, dysphoria, anxiety, motor agitation, aggression, hostility, euphoria, disinhibition, irritability, apathy, and other behavioral disturbances.

In another study that compared all available interventions for BPSD, it was found that pharmacological treatment with both risperidone and aripiprazole are superior to nonpharmacological treatment showing significant efficacy on CMAI (Jin and Liu, 2019). Some evidence suggests that safety concerns in the elderly population may be less prevalent with lower doses (0.25-2mg/day) of risperidone over a short-term period of 6–12 weeks (Oshima, 2008; Ballard et al., 2009; Torres-Lista et al., 2019). Following a review of clinical studies, on January 1, 2020, the Australian Pharmaceutical Benefits Scheme recommended that the use of risperidone should be limited to 12 weeks (National Prescribing Services, 2020).

## Regulatory Approvals and Off-Label Use

The rate of off-label use of antipsychotics worldwide is still high (Kirkham et al., 2017). Risperidone is reportedly the antipsychotic the most commonly prescribed off-label (Leslie et al., 2009; Leslie and Rosenheck, 2012). This can be partly explained by the fact that the US FDA has not yet approved any medication for treating BPSD (Maher et al., 2011). Despite clinical evidence supporting the efficacy of antipsychotics in the management of BPSD, so far, safety concerns appear to prevent FDA approval. Warnings started in 2002 with Health Canada advising physician to assess the risks and benefit of antipsychotic drugs in elderly patients and to immediately report signs and symptoms of CVAE (Health Canada Therapeutic, 2002). The FDA followed with warnings of increased CVAE for risperidone in April 2003 and for aripiprazole in February 2005. In addition, in April 2005, the FDA issued a health advisory warning of an increased risk for death with atypical antipsychotics in persons with dementia (Schneider et al., 2005; US Food Drug and Administration, 2005).

Despite safety concerns, risperidone remains a popular therapeutic choice for patients with Alzheimer’s disease and behavioral symptoms, especially those with more severe agitation and aggressive behaviors and has been approved for this indication in many countries (McNeal et al., 2008). Indeed, in 2008, the European Union approved risperidone for the short-term for up to 6 weeks management of persisting and severe aggression in individuals with Alzheimer’s disease who have failed nonpharmacological treatment. Health Canada and The Australian’s Therapeutic Goods Administration (2020) who had previously approved risperidone for behavioral disturbances in dementia reviewed this indication in 2015 following the safety issues and restricted risperidone’s indication for severe dementia of the Alzheimer type. It should be noted that risperidone is the only antipsychotic approved for the treatment of severe BPSD despite positive clinical evidence for other antipsychotics such as aripiprazole and quetiapine. This may be partly because risperidone is the oldest atypical antipsychotic on the market after clozapine and has well established use.

While risperidone is currently the only atypical antipsychotic approved in some countries for the treatment of BPSD, it is worthy to note that, another drug, pimavanserin, a selective serotonin-2A (5HT2A) receptor inverse agonist and already approved by the US FDA for Parkinson’s disease-related psychosis (PDP) is currently under development for dementia-related psychosis after a favorable phase II clinical trial result (Hacksell et al., 2014; Ballard et al., 2018). It should be noted that the effectiveness of pimavanserin in PDP was demonstrated at 4–6 weeks; however, there is no robust data on the onset of efficacy of the off-label use of risperidone in PDP (Cummings et al., 2014). An international Delphi consensus formed to prioritize existing and emerging treatments for BPSD placed a priority for risperidone for existing treatments and gave the greatest priority for future treatments to pimavanserin (Kales et al., 2019). A recent topline result from the pivotal phase III trial (the HARMONY trial, ClinicalTrials.gov number: NCT03325556) suggested that, in patients with dementia-related psychosis, pimavanserin reduced the risk of relapse of psychosis by 2.8 fold in comparison to placebo (Acadia Pharmaceuticals, n.d.). It remains to be seen whether this positive finding will pave a way for pimavanserin to secure a regulatory approval and subsequently become more favorable than risperidone.

## Conclusion

In patients with BPSD, treatment choices should be based on a positive risk-benefit ratio. Given the current evidence on the clinical effectiveness and safety of risperidone in the management of BPSD, its use should be restricted to patients with severe symptoms (aggression, agitation, or psychosis) who fail to respond adequately to nonpharmacological treatments. In this case, a low dose (0.25-2 mg daily) and short treatment duration (6-12 weeks) must be favored. Moreover, risperidone must be avoided in patients with a history of CVAE or with risk factors for stroke. Clinicians should also monitor patients for parkinsonism and risk of fall, using a fall rating scale. Risperidone should be stopped after 12 weeks if the risk of adverse events increases, or no benefit is observed.

Consistent with the best practice, before clinicians consider prescribing risperidone to patients with BPSD, the implementation of DICE (describe, investigate, create, and evaluate) approach should come first (Kales et al., 2015). In this approach, clinicians, and caregivers can better identify patients who might benefit from a pharmacological treatment. Since the US FDA has not yet approved any medication for BPSD and risperidone is the only approved drug for BPSD in some regions/countries (Europe, Australia, Canada, and the United Kingdom), research on other more effective and safer alternatives for BPSD is highly needed.

## Author Contributions

IY and MH determined the outline, reviewed the literature, wrote, and approved the manuscript.

## Conflict of Interest

The authors declare that the research was conducted in the absence of any commercial or financial relationships that could be construed as a potential conflict of interest.
